# Overexpression of *Arabidopsis thaliana ERI*, the homolog of *C. elegans Enhancer of RNAinterference*, leads to enhanced growth

**DOI:** 10.3389/fpls.2015.00531

**Published:** 2015-07-22

**Authors:** Rhonda C. Meyer, Gunnar Hönig, Ronny Brandt, Fernando Arana-Ceballos, Cathleen Neitsch, Gunter Reuter, Thomas Altmann, Markus Kuhlmann

**Affiliations:** ^1^Department of Molecular Genetics, Leibniz Institute of Plant Genetics and Crop Plant Research (IPK Gatersleben), Stadt Seeland, Germany; ^2^Department of Developmental Genetics, Institute of Biology, Martin Luther University Halle-Wittenberg, Halle (Saale), Germany

**Keywords:** ERI, Enhancer of RNAi, small RNA, *Arabidopsis*, biomass, enhanced growth

## Abstract

Organisms adopt a wide range of strategies to adapt to change. Gene silencing describes the ability of organisms to modulate the expression of susceptible genes at certain times at the transcriptional or the translational level. In all known eukaryotic organisms 21-nt long short interfering RNAs (siRNAs) are the effector molecules of post-transcriptional gene silencing (PTGS), while 24-nt long siRNAs are involved in PTGS in plants. Mutant studies in *Caenorhabditis elegans* lead to the identification of the enzyme ERI (Enhancer of RNAinterference) with enhanced PTGS. Although the genes involved in growth vigor and growth rate are still unknown, it becomes clearer that the population of small RNAs plays a role in the very early phase of plant development. To pinpoint the link between growth and siRNAs, the expression of *Arabidopsis* uni-gene *Enhancer of RNAi* (*ERI*) homolog from *C. elegans* was modulated. Increased degradation of small RNAs was achieved by ectopic *AtERI* overexpression *in planta*. Based on global small RNA analysis, *AtERI* overexpression affects mainly the population of 21 mers, excluding miRNAs. To identify target genes, AtERI gain-of-function mutants were analyzed, and differentially abundant small RNAs were identified. Plants with an elevated level of *AtERI* were bigger in all three light intensities analyzed, indicating an inhibitory function of particular small RNAs in plant growth, with differences in relative growth rates depending on developmental stage and light intensity. Understanding the role of these siRNAs could open new avenues for enhancing plant growth.

## Introduction

Gene silencing is a natural genetic mechanism that allows organisms to control gene expression according to developmental stage (Boerjan et al., 1994) or viral infections (Covey et al., 1997; Ratcliff et al., 1997). In *Arabidopsis thaliana* the mechanism of gene silencing is very well understood and the most important factors are identified (Bologna and Voinnet, 2014). In these mechanisms small RNAs are the most important molecules determining the silencing target specificity by their sequence homology. Based on their function and processing pathway, small RNAs are grouped into microRNAs (miRNAs, Axtell, 2013) and small interfering RNAs (siRNAs, Le Trionnaire and Twell, 2010). miRNAs are processed from hairpin forming, self-pairing single stranded precursor molecules. The length of miRNAs varies from 18 to 24 nt. The most abundant families are 21-nt long (Cuperus et al., 2010). siRNAs are the effector molecules of gene silencing. They are processed from double-stranded precursor RNAs. While 21-nt long siRNAs are involved in post-transcriptional gene silencing (PTGS), the presence of 24-nt long siRNAs is a typical hallmark for transcriptional gene silencing (TGS, Matzke et al., 2007) in plants. These heterochromatic RNAs are guiding the RNA-directed DNA methylation (RdDM) machinery in the nucleus to perform *de novo* DNA methylation at complementary genomic locations. An additional class of 22-nt long small RNAs, which is not related to gene silencing, is derived from degraded chloroplastic transcripts and results from the protective action of pentatricopeptide repeat (PPR) proteins against exonucleases (Ruwe and Schmitz-Linneweber, 2012).

### Role of Small RNAs in Development

The role of miRNAs in plant development is currently a point of investigation. The involvement of miRNAs as key regulators of flowering time (miR159, miR172, miR156, and miR171), hormone signaling (miR159, miR160, miR167, miR164, and miR393), or shoot and root development (miR164), was reviewed by (Wang and Li, 2007). During early seedling development the regulation mediated by the presence of miR165, miR166, miR164, and miR319 is of special importance for germination and developmental phase transitions (Wang and Li, 2007; Rubio-Somoza and Weigel, 2011). miR396 was identified as a regulator of the family of GRF transcription factors. Ectopic overexpression of this miRNA resulted in altered leaf shape and decreased cell number of the leaves (Liu et al., 2009a). The degradation of miRNAs was associated with the enzymatic activity of SDN1 (Small RNA degrading nuclease 1, Ramachandran and Chen, 2008). SDN1 was shown to be deterred by targets with 2′-*O*-methyl modification on the 3′ terminal ribose of single-stranded siRNA *in vitro*.

As the class of siRNAs is more inhomogeneous with respect to their processing and function, the investigation of the involvement of particular siRNAs is more complex. The involvement of siRNAs during early plant development has been described for at least two mechanisms. The first one involves PTGS via the presence of 21-nt long small RNAs (Mallory and Vaucheret, 2010). The second mechanism involves 24-nt heterochromatic small RNAs and addresses gene regulation mediated by RNA directed DNA methylation (Matzke et al., 2007; He et al., 2011). Particular 24-nt long siRNAs might have an impact on the early growth vigor of *Arabidopsis* plants: A decrease in the amount of 24-nt long small RNAs correlated with an increase in biomass during the early growth phase [10–14 days after sowing (DAS), Groszmann et al., 2011]. This correlation implies a functional relevance of 24-nt long small RNAs for hybrid incompatibility as well as interspecific hybrids (Ng et al., 2012). An analysis of hybrid crosses between the accessions Landsberg *erecta* and C24 revealed the importance of the class of heterochromatic 24 mers that are associated with the RdDM mechanism. In a genome-wide study of 24 mers and DNA methylation, candidate genes could be identified that are differently methylated in the offspring compared to their parents upon a hybridisation event (Shen et al., 2012). From that analysis 77 genes were identified as being susceptible to differential DNA methylation in the hybrids. The same correlation of heterochromatic small RNAs and improved growth vigor could also be detected in hybrid crosses of wheat, rice (He et al., 2010) and maize (Barber et al., 2012; He et al., 2013). The analysis of the *mop1* mutant, affecting the homolog of *RDR2*, an RNA-dependent-RNA-polymerase involved in the production of silencing related small RNAs (Jia et al., 2009), revealed that in parallel to the reduced 24-nt heterochromatic small RNAs an increase of 22 and 21-nt small RNAs was detectable in maize (Barber et al., 2012). Crosses derived from *mop1* also showed better performance in corn yield.

### *ERI* (*Enhancer of RNAi*) Encodes a 3′-5-Endonuclease Belonging to the Ribonuclease H-like Protein Family

Although the DICER mediated processing of small RNA is well understood, no candidate gene involved in the degradation or further processing of siRNAs has been described in plants so far. The first enzyme reported to be involved in siRNA degradation was ERI-1 (ENHANCER OF RNAi) isolated in *C. elegans* based on its effect on RNAi (Kennedy et al., 2004). The contribution of this enzyme to the antiviral defense mechanisms of *C. elegans* was demonstrated (Wilkins et al., 2005). While in *eri-1* mutant cell culture vesicular stomatitis virus accumulation was reduced, the accumulation of virus in single cells was increased. *In vitro* analyses using recombinant 3′hEXO, a human homolog of *C. elegans* ERI-1, revealed that this enzyme degrades the 3′ overhangs of siRNAs, while the double-stranded region remained unaffected (Yang et al., 2006). Additional ERI-homologs are described in *Schizosaccharomyces pombe* (Gabel and Ruvkun, 2008), *Mus musculus* (Hong et al., 2005), and *Dictyostelium discoideum* (Kuhlmann et al., 2005). Based on sequence similarities the coding region of *At3g15140* was identified as *ERI-1* homolog in *A. thaliana* (Ramachandran and Chen, 2008).

We analyzed the effects of the ectopic overexpression of the *Arabidopsis Enhancer of RNAi* (*ERI*) homolog to verify its role in degradation of siRNAs in *Arabidopsis*, to identify target genes undergoing PTGS, and to elucidate the link between growth and siRNAs.

## Materials and Methods

### Plant Material

The *35S:AtERI* lines were generated using a pCAMBIA1302 binary vector backbone which carried the *At3g15140* derived cDNA under control of the *CaMV35S* promoter. The *ERI-1* cDNA was amplified from RNA of accession Col-0 after reverse transcription (RevertAid Reverse Transcriptase, Thermo) using primers ERI full for and rev (Supplementary Table). The cDNA was cloned via the pSC-A vector using a Strataclone PCR-cloning kit (Agilent Technologies, cat.no. 240205) and constructed into pCAMBIA1302 using SpeI restriction site, replacing the GFP. Transformation of *A. thaliana* Col-0 by the floral dip method (Clough and Bent, 1998) was performed, using *A. tumefaciens* C59 pGV2260. The resulting plants were selected for hygromycin (20 mg/l) resistance. Single-copy T-DNA insertions were identified by segregation and Southern blot analysis. The integrity of the transferred transgene was analyzed by sequencing of PCR products. The insertion site of the line that was used as crossing partner was estimated by Genome walker 2.0 kit (Clontec). The insertion of the T-DNA was identified within a repetitive genomic region (SINE9) and did not affect any gene function. For antibiotic resistance test of plants on selective tissue culture medium, seeds were surface-sterilized (10 min, 8% NaClO) and grown under long day regime on hygromycin (20 mg/l; *35S:AtERI*). To monitor PTGS the *35S:AtERI* was crossed to a reporter line containing 4 copies of β-*GLUCURONIDASE* (GUS, Schubert et al., 2004). The homozygous plants identified by PCR and segregation analysis containing reporter line *4xGUS* and *4xGUS*/*35S:AtERI* were used for high throughput sequencing.

For the analysis of plant growth, seeds of the selected homozygous *35S:AtERI* line and the wild type Col-0 accession were stratified before germination for 3 days at 4° C in the dark. Plants were cultivated on soil at 21° C under a 16 h light/8 h dark (long day) regime for seed production or under 8 h light/ 16 h dark (short day) regime to generate material for RNA and DNA analysis.

### RNA Analysis

Total RNA samples for transcript measurements were extracted from seedlings grown for 1 week on soil under short day conditions and from rosette leaves of plants grown for 6–8 weeks under short day conditions. Total RNA was extracted with Qiagen RNAeasy kit and small RNA was extracted from 1 g leaf tissue with the mirVana miRNA Isolation kit (Ambion) according to manufacturer’s protocol. cDNA was prepared using RevertAid H Minus M-MuLVRT (Fermentas) according to manufacturer’s protocol. mRNA analysis was performed on oligo dT transcribed cDNA using specific primers (Supplementary Table 1). cDNAs were quantified by the RT-qPCR method using the iCycler (Bio-rad) and the iQ SyBRGreen Supermix (Bio-rad). Program: 1: 5′ 95° C; 2: 15′ 95° C; 3: 30′ 65° C; 4: 30′ 72° C; 5: goto2 40x; 6: Melting curve 65° C 10′ +0.5° C 60 repeats; 7: 4° C in triplicate from two biological replicates. Mean values are indicated as bar heights, standard deviation of values as error bars. *PHOSPHOFRUCTOKINASE* (*PFK*; *AT4G04040*) and *ACTIN2* (*AT3G18780*, Supplementary Figure 1) was used as reference gene.

### High Throughput Sequencing of Small RNA

The fraction of small RNA (mirVana) was treated according the manufacturer’s protocol for Illumina TruSeq smallRNA sample prep kit. The size selection was performed on a 2% Agarose-TAE-Gel (2 h at 120V). The region of 135–170 bp was cut from the gel (mean fragment length wild-type: 136 bp, *35S:AtERI*: 137 bp, reference 50 bp-Ladder ThermoScientific) and purified with Qiagen PCR purification kit. Adapter trimming was performed via CLC Genomics Workbench (CLC Genomics Workbench 6.5.1, 2014) on the 3′ end of the read with the P8 adapter 5′—TGGAATTCTCGGGTGCCAAGGAACTCCAGTCAC-Index-ATCTCGTATGCCGTCTTCTGCTTG—3′. After trimming the reads were mapped on the *A. thaliana* reference genome extracted from TAIR10 (Lamesch et al., 2012) with segemehl (Hoffmann et al., 2009) using standard parameters. The resulting SAM-Files (Li et al., 2009) were processed with a custom made Perl-script (URL^1^) and the extracted read count and coverage normalized with counts per million reads. All plots were produced with R (R Development Core Team, URL^2^).

### Plant Growth Assays

Plants were grown in a mixture of 85% (v) red substrate 2 (Klasmann-Deilmann GmbH, Geeste, Germany) and 15% (v) sand in 96-well-trays (QuickPot QP 96T, HerkuPlast Kubern GmbH, Ering, Germany). After 2 days of stratification at 5° C in constant darkness, seeds were germinated and seedlings cultivated in a walk-in growth-chamber with a 16/8 h day/night regime, 20/18° C, 60/75% relative humidity. To avoid position effects, trays were rotated around the growth chamber every day. Plants were grown at 51, 101, or 187 μmol m^–2^s^–1^ photosynthetically active radiation (PAR) in a lattice square design in two independent experiments with 32, respectively 24, replicates per light condition.

### Determination of Leaf Area and Shoot Dry Biomass

For seed size determination, 20 *Arabidopsis* seeds were fixed with adhesive plate seal (Thermo Fisher Scientific, Loughborough, UK) on a sheet of paper displaying a 5 cm scale bar. The seed were scanned at a resolution of 1200 dpi on an Epson Expression 10000XL flatbed scanner (Seiko Epson Corporation, Suwa, Japan). The measurement of seed length, width and area was performed using the Evaluator software (developed by Dmitry Peschansky, IPK Gatersleben) according to the software instructions. The Evaluator algorithm isolates the seed area from the background based on differences in pixel intensities, creates a contour boundary and counts the pixels inside the boundary as a measure of area (Meyer et al., 2012).

Images of plants were taken until 16 DAS and whole leaf area was determined using the GrowScreen imaging system and software described in (Walter et al., 2007). Leaf area was extracted from the images using the software Bayer2Area (Meyer et al., 2010).

Shoot dry biomass was determined 20 DAS. The harvested aerial parts of the plants were placed in a vacuum oven at 80°C for 48 h. Dry biomass was measured using an analytical balance (Excellence XS205 Dualrange, Mettler Toledo, Gießen, Germany) with LabX direct Balance software. Mean shoot dry biomass in mg plant^–1^ and mean leaf areas in mm^2^ plant^–1^ were estimated using a nested two-factorial ANOVA with line and light-intensity as independent variates and seed size as covariate.

Relative growth rates (RGRs) were calculated as [LN(leaf area at timepoint 2)–LN(leaf area at timepoint 1)]/(timepoint 2–timepoint 1). Differences in RGR were tested via a two-factorial ANOVA with natural log-transformed leaf area as the dependent variable (Poorter and Lewis, 1986). Seed size was taken as leaf area at timepoint t_0_. The interaction term between line and time was partitioned using a second-order polynomial contrast for the factor time. According to Poorter and Lewis (1986), a significant linear interaction term indicates that differences in RGR are linear over time, i.e., maintained during the experiment, while the quadratic interaction term measures the extent to which differences in RGR changed with time.

## Results

### Identification and Characterization of the *Arabidopsis thaliana* ERI Homolog

**FIGURE 1:**
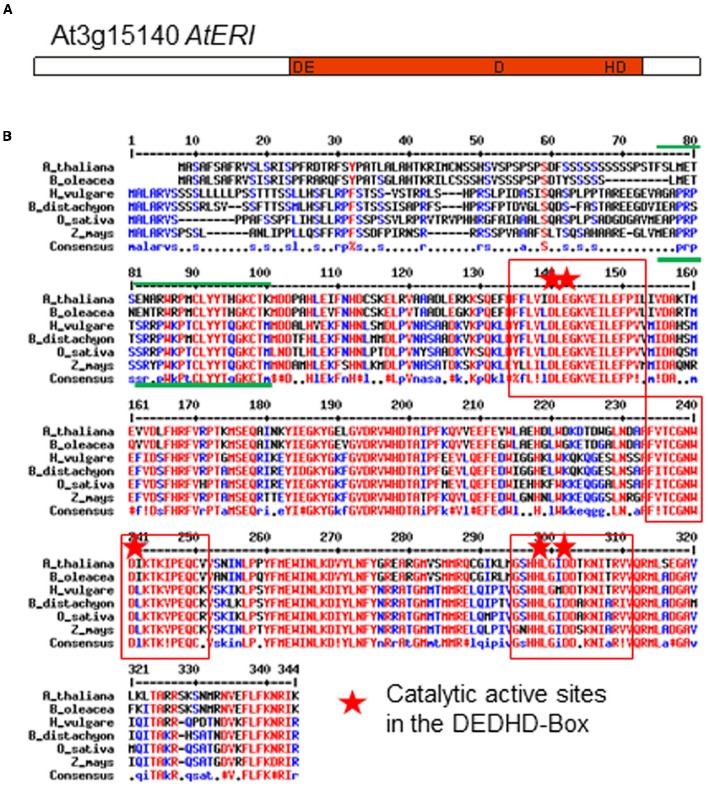
**(A)** Structure and **(B)** Comparison of *Arabidopsis* and other crop plant ERI proteins (*Brassica oleracea, Hordeum vulgare, Brachypodium distachyon, Oryza sativa, Zea mays*). Red letters: identical amino acids, red squares: regions in the exonuclease-domain (DEDHD) of high similarity, including the active catalytic sites (red star). Green lines indicate the SAP-domain.

*AtERI* (*At3g15140*) encodes a protein of 337 amino acids of the ribonuclease H-like superfamily. The catalytic core component of all enzymes belonging to the ERI subfamily is the DEDDh domain. This C-terminal domain (Figure 1A) is responsible for the 3′ overhang modifying activity on small RNA molecules. In addition the proteins from that subfamily contain a SAP domain (after SAF-A/B, Acinus, and PIAS), responsible for an interaction with nucleic acids (Aravind and Koonin, 2000). The SAP domain, which is located in the N-terminal part of the human, mice, worm and slime mold homologous protein, can also be found in the ERI-homologs from different crop plants such as *Brassica oleracea*, *Hordeum vulgare*, *Brachypodium distachyon*, *Oryza sativa*, and *Zea mays* (Figure 1B), indicating an evolutionary conservation in the plant kingdom. As the presence of SAP and tripartite DEDDh domains is unique to ERI proteins as compared to other exonucleases (Ramachandran and Chen, 2008), *Arabidopsis At3g15140* was identified as the only putative *ERI-1* homolog.

The full-length cDNA derived from *At3g15140* contains six exons. Notably, the first exon contains a TCT-microsatellite structure (starting 226 bp after ATG) which varies in length in different *Arabidopsis* accessions. Based on sequence complementarity, it is a *miR5021*-cleavage target site (RegRNA2.0, Chang et al., 2013).

According to expression databases (Genevestigator, Zimmermann et al., 2004, and eFP browser, Winter et al., 2007), expression of *At3g15140* is relatively weak, with peaks of expression in the early development from seeds to cotyledons, and during the transition from vegetative to reproductive stage.

**FIGURE 2:**
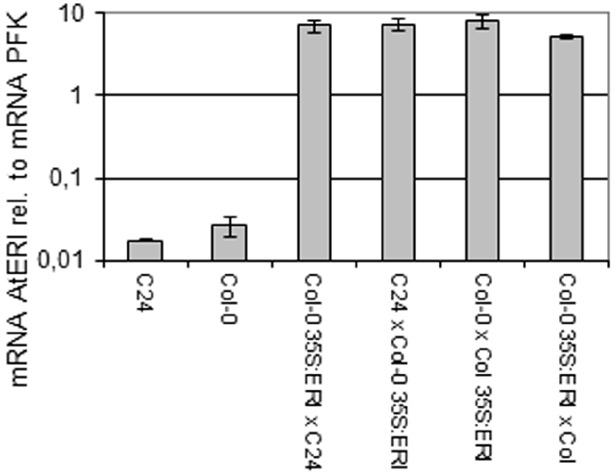
**RT-qPCR analysis of *AtERI* transcript relative to *PFK* mRNA in leaves.** mRNA level of AtERI in leave RNA relative to mRNA of *PFK* (*AT4G04040*), *N* = 5, Errorbars indicate Standard deviation.

Plants expressing the *AtERI* cDNA under the control of the *CaMV35S* promoter were generated by *Agrobacterium*-mediated transformation. Three independent plant lines were obtained after transformation. The presence of a single T-DNA integration locus in the genome was concluded by genetic 3:1 segregation on hygromycine selection. The mRNA level of endogenous and transgenic *AtERI* was quantified by qRT PCR relative to *ACTIN2* mRNA in leaves of 6 week old soil grown plants. The homozygous plant line with the high expression (approximately 850-fold more *AtERI* transcripts in leaves than the *Col-0* wild-type plants) was selected for further studies and crossed to Col-0 and C24. Expression of *AtERI* was tested in the offspring (Figure 2, Supplementary Figure 1). The *35S::AtERI* line showed no obvious phenotypical differences in morphology and onset of flowering within short and long-day regimes when compared to wild-type plants (data not shown).

### Sequencing of Small RNA Reveals a Reduction of 21 mers in *AtERI* Overexpression Plants

**FIGURE 3:**
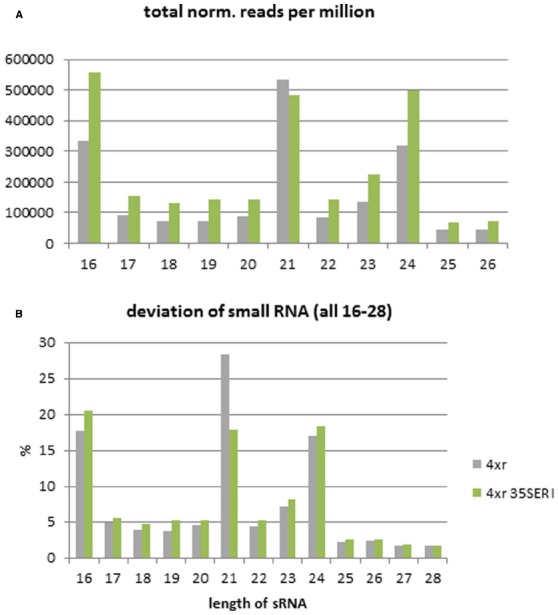
**Size distribution of small RNAs. (A)** Read count obtained by high throughput sequencing normalized to reads per million. **(B)** Population of small RNAs separated in size classes given in percent of total estimated reads from Col-0 and *35S:AtERI* in 4xGUS reporter background. 4xr (gray): Col-0 with 4xGUS reporter, 4xr 35SERI (green): *35S:AtERI* with 4xGUS reporter.

**FIGURE 4:**
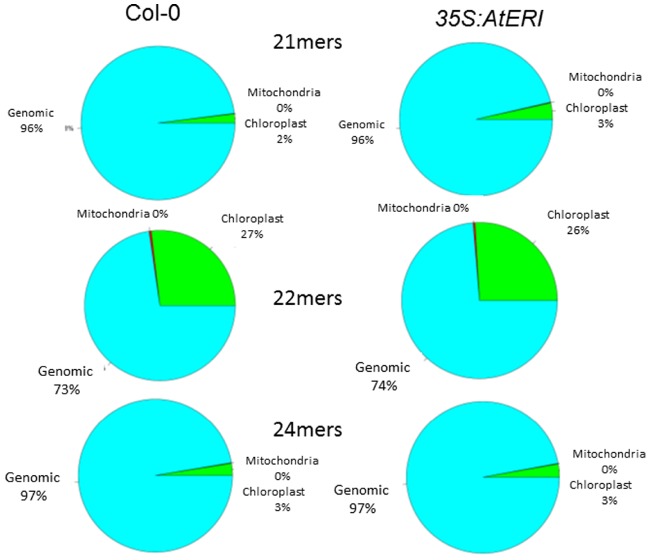
**Abundance of chloroplast derived small RNAs is not altered due to *AtERI* overexpression.** Obtained reads of Col-0 (left) and *35S:AtERI* (right) in 4xGUS reporter background were selected based on their size and mapped to the nuclear (blue), chloroplast (green) and mitochondrial (red) genome of *Arabidopsis thaliana*.

Leaves of Col-0 wild type and *35S:AtERI* overexpression plants containing a PTGS reporter system (Schubert et al., 2004) were used for RNA extraction. The enriched fraction of small RNAs was subjected to high throughput sequencing utilizing the Illumina TruSeq smallRNA-Kit and Ilumina HiSeq 2000 sequencer. After trimming of adapter sequences and selecting molecules in the range from 16 to 32 nt, 5.4 million sequences from the wild type and 9.5 million sequences from the *35S::AtERI* line were used for mapping to the *Arabidopsis* genome and the miRNA database (miRBASE, Kozomara and Griffiths-Jones, 2014). For analysis of the relative abundance, obtained data were normalized to reads per million and the relative abundance of different size classes was estimated (Figure 3). From the distribution of the read sizes, it is apparent that the class of 21-nt long reads is underrepresented in the *AtERI* overexpressing line compared to the wild type Col-0 (17.8% versus 28.4%). This is consistent with the proposed function of AtERI as siRNA specific exonuclease reported from other organisms. The reads were mapped to the *Arabidopsis* genome (TAIR10) and separated according to their best match into nuclear, chloroplast and mitochondrial genome origin. No significant change in the relative abundance of small RNAs originating from the chloroplastic or the mitochondrial genome was detected (Figure 4). Neither 21 mers nor 24 mers related to gene silencing, homologous to chloroplast genes, nor the 22 mers derived from degradation events (Ruwe and Schmitz-Linneweber, 2012) showed a differential abundance. Among the list of genes with differential abundant small RNAs only one gene encoded by the chloroplast genome ATCG00620 could be identified. From the results obtained and the absence of any phenotypic alteration of the leaves, a leaf specific function of AtERI involved in degradation of small RNAs of chloroplastic origin was excluded. Furthermore the absence of any small RNAs homologous to the CaMV35S promotor sequence was indicative for the absence of any trans-silencing event, based on presence of multiple promotor copies (Supplementary Figure 1B).

For analysis of miRNA, the identified reads were mapped to the list of published miRNA sequences (Kozomara and Griffiths-Jones, 2014). Reads were normalized (reads/million) and evaluated for differential abundance with CLC workbench software (QIAGEN). 159 sequences could be assigned to *Arabidopsis* miRNAs, while 34 sequences showed homologies to miRNAs from other species. Comparing the relative abundance of all miRNAs analyzed we found 8 miRNAs less abundant in the *AtERI* overexpressing plants (with miR841 showing the strongest decrease by 12-fold) and 94 miRNAs without significant influence. For miR841 no function or downstream target gene is known. 84 members of miRNA-families are more abundant in the *AtERI* overexpressing plants (Supplementary Table 2). Among all miRNA sequences analyzed, an increased abundance of miRNAs normalized to reads per million could be found in the *AtERI* overexpressing plant. Notably, miRNA396 (ath-miR396a-3p), reported to suppress GROWTH-REGULATING FACTOR (Liu et al., 2009b; Debernardi et al., 2012) showed a 2.8-fold increase in normalized reads compared to Col-0. The detected general increase of miRNAs resulted from the proportional decrease of highly abundant 21 mers in the *AtERI* overexpressing plant. No general influence of overexpressed *AtERI* was found on miRNA processing.

In order to identify target genes undergoing PTGS, the 21-nt long reads corresponding to siRNAs (Martinez de Alba et al., 2013) were mapped to the coding regions of the genes. The presence of 21 mers homologous to the coding regions is indicative of PTGS. The opposite trend compared to the global abundance of 21 mers was observed. 116 regions/genes with higher abundance in the *AtERI* overexpressing plant could be identified (Supplementary Table 3). For functional categorization of the target regions/genes with higher abundance of small RNA in the AtERI overexpressing line, the MAPMAN software (Thimm et al., 2004) was used. The gene ontology mapper clearly shows the high abundance of small RNAs associated with genes from the category “RNA.” The genes in this category are either encoding tRNAs or tRNA-related proteins.

In contrast, only five coding regions/genes were found to be associated with siRNAs downregulated in the *AtERI*-overexpressing line (Table 1). Targets being less subjected to PTGS include the genes encoding growth regulatory factors GRF3 (*AT2G36400*) and GRF4 (*AT3G52910*). Also a small nuclear non-coding RNA (*AT1G26235*, Marker et al., 2002), a gene encoding a chloroplast located pentatricopeptide repeat-containing protein (SVR7) involved in chloroplast biogenesis via RNA binding (*AT2G17033*, Liu et al., 2010) and a not yet characterized gene (*AT1G47389*) could be identified.

**Table 1:** **List of annotated genes with less association of 21 mers in 35S:AtERI**.

**Gene**	**logFC**	**logCPM**	**PValue**	
AT2G36400	–2,02621676	3,62198957	0,00051627	GRF3 (AT2G36400) growth-regulating factor 3
AT3G52910	–2,05526714	3,61842118	0,0004839	GRF4 (AT3G52910) growth-regulating factor 4
AT2G17033	–2,26204171	2,19855313	0,00125456	Pentatricopeptide repeat-containing protein
AT1G26235	–4,79504295	0,43524652	0,00061644	ncRNA
AT1G47389	–7,5390791	0,74908175	0,00017327	Uncharacterized protein

### Increased Growth in AtERI Overexpressing Plants

Growth differences between Col-0 and the *AtERI* overexpressing line were analyzed on two levels: static differences at harvest as biomass at 20 DAS, and dynamic differences during development (0–16 DAS) as RGR based on leaf area.

The *AtERI* overexpressing line had significantly (*p* < 0.001) higher aerial biomass under all light conditions (Table 2). Differences in leaf area depended on the developmental stage only at low light intensity (Table 2).

**Table 2:** **Biomass and leaf area of 35S:AtERI and Col-0**.

**Trait**	**Light intensity (μ mol m^–2^s^–1^)**	**Col-0**	**35S:AtERI**	***p*-value**	**Significance (Bonferroni)**	**ese**
Biomass						
20DAS (mg plant^–1^)	51	0.940	1.110	< 0.001	***	0.2386
	101	4.973	6.840	< 0.001	***	
	187	10.196	14.378	< 0.001	***	
Leaf area						
6DAS (mm^2^ plant^–1^)	51	1.824	2.038	0.050	ns	0.1516
	101	3.569	4.626	<0.001	***	
	187	3.994	5.730	<0.001	***	
Leaf area						
8DAS (mm^2^ plant^–1^)	51	3.91	4.1	0.418	ns	0.3830
	101	9.12	11.79	<0.001	***	
	187	11.65	16.06	<0.001	***	
Leaf area						
14DAS (mm^2^ plant^–1^)	51	19.9	22.3	0.007	**	2.980
	101	63.8	85.9	<0.001	***	
	187	94.1	130.9	<0.001	***	
Leaf area						
16DAS (mm^2^ plant^–1^)	51	33.9	40.0	<0.001	***	6.340
	101	128.2	182	<0.001	***	
	187	202.2	273.9	<0.001	***	

Values represent mean values estimated with 2-catorial ANOVA; ese indicates the estimated standard error of means within the same light condition. ns, not significant; ^**^p < 0.01, ^***^p < 0.001.

**FIGURE 5:**
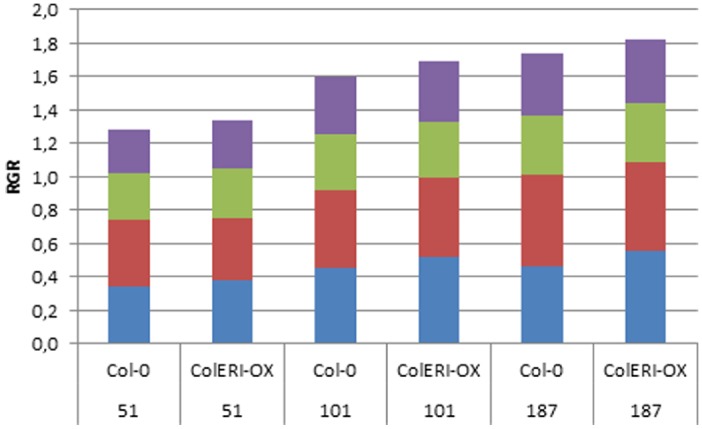
**Relative growth rates in Col-0 and *35S:AtERI* at different light intensities.** RGRs were estimated from seed (0 DAS) and leaf area (6, 8, 14, 16 DAS) and are given in mm^2^ d^–1^: blue, RGR0-6 (between 0 and 6 DAS); red, RGR6-8; green, RGR8-14; purple, RGR14-16. 51, 101, 187 indicate the light intensities (μmol m^–2^s^–1^) the plants were grown in.

To detect possible differences in the developmental pattern, RGRs between 0, 6, 8, 14, and 16 DAS were determined based on leaf area. Differences between RGRs were estimated by a two-factorial ANOVA (Table 3). Only the quadratic interaction term was significant, i.e., differences in RGRs changed over time (Poorter and Lewis, 1986). The overexpressing line showed overall higher RGR than the Col-0 wild type (Figure 5). The largest differences occurred for all light intensities during early growth (RGR_0–6_), with the overexpressing line displaying higher values. Interestingly, ectopic overexpression of *AtERI* also led to higher RGRs at the later developmental stage (RGR_14–16_), except at 187 μmol m^–2^s^–1^.

**Table 3:** **Differences between relative growth rates (RGRs)**.

**Source of variation^a^**	**s.s.^b^**	**d.f.^c^**	***p*^d^**
Light	396.901	2	<0.001
Line	9.09441	1	<0.001
Time	7859.79	4	<0.001
Light.Line.Time: linear	0.24859	2	0.165
Light.Line.Time: quadratic	0.64553	2	0.009
Residual	99.9416	1450	
Total	8225.03	1497	

^a^Sources of variation comprise the independent variables “light” (the different light intensities), “line” (Col-0 and 35S:AtERI) and “time” (time points of leaf area determination), and interaction terms. Only the values for the interaction term Light.Line.Time are shown.

^b^Sum of squares.

^c^Degrees of freedom.

^d^Probability of the F test.

## Discussion

### The Reduction of siRNAs in AtERI Overexpressing Plants Supports the Proposed Function of Degradation

The plants used for the present study are ectopically overexpressing the *A. thaliana* homolog of the 3′-5- endonuclease Enhancer of RNAi (*AtERI*) under control of the promotor of the Cauliflower mosaic virus (*ProCaMV35*). The enhanced ectopic expression of *AtERI* was confirmed by the increased presence of *AtERI* mRNA. Based on the homology and domain structure of AtERI it was concluded that the function of AtERI is similar to the already described exonucleases from human, mice, worm and slime mold. The described protein structure (Figure 1) is conserved among all crop species analyzed and is indicative of a unique and highly conserved function in the plant kingdom. As described in *C. elegans*, the function of ERI could be addressed to degradation of small RNAs by removal of the protruding 3′-nt. This shortening of siRNA led to their exclusion from the mechanism of PTGS.

The profile of small RNAs obtained by high throughput sequencing showed a reduction of sequences with the specific size of 21-nts. This is in agreement with the proposed function of AtERI as 3′-5-endonuclease. Also the increased abundance of 16-nt long small RNA in the AtERI overexpressing plant argues for an increased degradation process accumulating the smaller products. Mapping of the small RNAs to the miRBASE and the *A. thaliana* coding regions reveal the specificity of AtERI function on 21 mer sized siRNAs. As the majority of miRNAs are unchanged in their abundance it can be concluded, in agreement with already published data (Ramachandran and Chen, 2008), that the degradation of miRNAs is performed by SDN1 and not affected by ERI.

The population of 22-nt small RNAs, mainly derived from incomplete degradation of chloroplast derived transcripts (Ruwe et al., 2011), is also not affected by ectopic AtERI overexpression. The functional contribution of SVR7, found to be a target less susceptible by increased AtERI expression to the pool of chloroplast derived small RNAs is not clear from the current state of knowledge.

Despite the global reduction of 21-nt sized RNAs, an increase of siRNA associated with the coding regions was detected. This is in agreement with the fact that the majority of targets subjected to PTGS is derived from retroelements and transcribed non-coding regions (Vaucheret and Fagard, 2001).

The increased abundance of 24 mer, triggers of the TGS mechanism and associated with RNA directed DNA methylation, supports the model for an already proposed backup system of retroelement inactivation (Creasey et al., 2014) in the AtERI overexpressing plant.

Summarizing the data it can be concluded that AtERI functions as 3′-5-endonuclease with specificity for siRNAs involved in the mechanism of PTGS in *A. thaliana*.

### Overexpression of *AtERI* Affects the Early Growth

Plants ectopically overexpressing *AtERI* accumulated more biomass than the Col-0 wild type. The differences were established early during development, most likely during seedling establishment. Small increases in RGRs between lines may lead to large differences in size (Milborrow, 1998). A larger leaf area during seedling growth would allow the *AtERI* overexpressing lines to absorb more light than the Col-0 wild type, potentially resulting in increased photosynthetic activity per plant. The transcription factor AtGRF3, for which small RNAs homologous to the coding region were identified, is involved in cell expansion during early development (Kim et al., 2003). This is also consistent with the native expression of *AtERI* during early development. Interestingly, native *AtERI* expression shows a second peak during the transition from vegetative to reproductive growth, which might explain the increased RGRs during the later development.

Although biomass differences between *AtERI* overexpressing and wild type plants were observed for all three light intensities analyzed, the temporal pattern of RGR differences varied with the light intensity. The stronger initial growth at the highest intensity was dampened by reduced growth at later stages. The reasons for this behavior remain unclear. It is well known that growth differences can be influenced by different light intensities. In F1 hybrids of the *Arabidopsis* accessions Col-0 and C24, biomass heterosis was increased in higher light intensities (240 vs. 120 μmol m^–2^s^–1^) due to a sustained increase in RGR (Meyer et al., 2004). Light intensity positively affected silencing initiation and spread in *Nicotiana* (Kotakis et al., 2010).

### Speculation About the Function of Identified Target Genes in Early Plant Growth

The mapping of differentially abundant 21-nt sized RNA to the coding region of the *Arabidopsis* genome led to the identification of 116 genes with increased abundance of siRNAs in the *35S:AtERI* plant. The presence of 21 mers as hallmark of PTGS indicates that these genes undergo increased silencing in the *AtERI* overexpressing plant compared to the wild type. Derived from the MAPMAN based gene ontology view, a substantial proportion of these genes are associated with RNA function. More important, several well characterized ontology classes connected with energy production and general biogenesis are strongly underrepresented. From this can be concluded that no specific enhancement of gene silencing affects physiologically important genes.

Within the list of genes with decreased abundance of siRNAs, two important growth regulating factors could be identified: GRF3 and GRF4. The encoding genes are located within QTL for biomass heterosis and vegetative growth on chromosome 2 and 3, respectively (Meyer et al., 2010).

GRF3 and GRF4 are members of a transcription factor gene family that was already described for promoting plant growth during early development (Kim et al., 2003). The expression of *AtGRF3* in leaves was restricted to the very early stages of emerging leaves, consistent with the observed differences during early development. The presence of small RNAs homologous to the coding region of GRF3 and GRF4 in the wild type plant is indicative of small RNA based regulation/silencing in the early growth phase. The absence of these small RNAs would lead to reduced silencing, therefore a stronger expression, and might thus lead to increased biomass during early development. Members of the GRF family are regulated by miR396 (Liu et al., 2009b). Ectopic overexpression of miR396 lead to reduced leaf cell number and altered leaf shape. Although we did not see a general change in the population of microRNAs in the *AtERI* overexpressing plant, sequences with similarity to miR396 were analyzed and quantified. While the abundance of 20 nt long sequences (exact miRNA sequence) are not significantly altered the number of shorter sequences is increased in the *AtERI* overexpressing plant. Therefore it can be speculated that the turnover of miR396 is increased based on increased abundance of AtERI. Such increased turnover might also have a positive effect on the GRF gene expression and thereby contributing to increased production of biomass in the early growth phase. Based on the unchanged amount of miR396 (exact sequence) an influence of *AtERI* overexpression on miRNA396 and subsequent regulation of the GRF transcription factor family can be excluded.

Based on the results presented a model is proposed regulating the accumulation of early biomass and growth rate via increased expression of *GRF3* and *GRF4*. While in the wild-type situation these genes are targets of post-transcriptional regulation no small RNAs are detectable in the *AtERI* overexpressing plants. Therefore it is concluded that increased degradation of PTGS associated small RNAs lead to a deregulation of naturally suppressed target genes. The described release affects positively the accumulation of early biomass and growth rate.

### Conflict of Interest Statement

The authors declare that the research was conducted in the absence of any commercial or financial relationships that could be construed as a potential conflict of interest.

## Footnotes

1 http://www.perl.org/docs.html

2 http://www.R-project.org

## References

[B1] Aravind L. Koonin E. V. (2000). SAP—a putative DNA-binding motif involved in chromosomal organization. Trends Biochem. Sci. 25, 112–114. 10.1016/S0968-0004(99)01537-6 10694879

[B2] Axtell M. J. (2013). Classification and comparison of small RNAs from plants. Annu. Rev. Plant Biol. 64, 137–159. 10.1146/annurev-arplant-050312-120043 23330790

[B3] Barber W. T. Zhang W. Win H. Varala K. K. Dorweiler J. E. Hudson M. E. (2012). Repeat associated small RNAs vary among parents and following hybridization in maize. Proc. Natl. Acad. Sci. U.S.A. 109, 10444–10449. 10.1073/pnas.1202073109 22689990 PMC3387101

[B4] Boerjan W. Bauw G. Van Montagu M. Inze D. (1994). Distinct phenotypes generated by overexpression and suppression of S-adenosyl-L-methionine synthetase reveal developmental patterns of gene silencing in tobacco. Plant Cell 6, 1401–1414. 7994174 10.1105/tpc.6.10.1401 PMC160529

[B5] Bologna N. G. Voinnet O. (2014). The diversity, biogenesis, and activities of endogenous silencing small RNAs in *Arabidopsis*. Annu. Rev. Plant Biol. 65, 473–503. 10.1146/annurev-arplant-050213-035728 24579988

[B6] Chang T. H. Huang H. Y. Hsu J. B. Weng S. L. Horng J. T. Huang H. D. (2013). An enhanced computational platform for investigating the roles of regulatory RNA and for identifying functional RNA motifs. BMC Bioinformatics 14(Suppl. 2):S4. 23369107 10.1186/1471-2105-14-S2-S4 PMC3549854

[B7] Clough S. J. Bent A. F. (1998). Floral dip: a simplified method for *Agrobacterium*-mediated transformation of *Arabidopsis thaliana*. Plant J. 16, 735–743. 10.1046/j.1365-313x.1998.00343.x 10069079

[B8] Covey S. N. Alkaff N. S. Langara A. Turner D. S. (1997). Plants combat infection by gene silencing. Nature 385, 781–782.

[B9] Creasey K. M. Zhai J. Borges F. Van Ex F. Regulski M. Meyers B. C. (2014). miRNAs trigger widespread epigenetically activated siRNAs from transposons in *Arabidopsis*. Nature 508, 411–415. 10.1038/nature13069 24670663 PMC4074602

[B10] Cuperus J. T. Carbonell A. Fahlgren N. Garcia-Ruiz H. Burke R. T. Takeda A. (2010). Unique functionality of 22-nt miRNAs in triggering RDR6-dependent siRNA biogenesis from target transcripts in *Arabidopsis*. Nat. Struct. Mol. Biol. 17, 997–1003. 10.1038/nsmb.1866 20562854 PMC2916640

[B11] Debernardi J. M. Rodriguez R. E. Mecchia M. A. Palatnik J. F. (2012). Functional specialization of the plant miR396 regulatory network through distinct microRNA-target interactions. PLoS Genet. 8:e1002419. 10.1371/journal.pgen.1002419 22242012 PMC3252272

[B12] Gabel H. W. Ruvkun G. (2008). The exonuclease ERI-1 has a conserved dual role in 5.8S rRNA processing and RNAi. Nat. Struct. Mol. Biol. 15, 531–533. 10.1038/nsmb.1411 18438419 PMC2910399

[B13] Groszmann M. Greaves I. K. Albertyn Z. I. Scofield G. N. Peacock W. J. Dennis E. S. (2011). Changes in 24-nt siRNA levels in *Arabidopsis* hybrids suggest an epigenetic contribution to hybrid vigor. Proc. Natl. Acad. Sci. U.S.A. 108, 2617–2622. 10.1073/pnas.1019217108 21266545 PMC3038704

[B14] He G. M. Chen B. B. Wang X. C. Li X. Y. Li J. G. He H. (2013). Conservation and divergence of transcriptomic and epigenomic variation in maize hybrids. Genome Biol. 14, R57. 10.1186/gb-2013-14-6-r57 23758703 PMC3707063

[B15] He G. M. Zhu X. P. Elling A. A. Chen L. B. Wang X. F. Guo L. (2010). Global Epigenetic and Transcriptional Trends among Two Rice Subspecies and Their Reciprocal Hybrids. Plant Cell 22, 17–33. 10.1105/tpc.109.072041 20086188 PMC2828707

[B16] He X.-J. Chen T. Zhu J.-K. (2011). Regulation and function of DNA methylation in plants and animals. Cell Res. 21, 442–465. 10.1038/cr.2011.23 21321601 PMC3152208

[B17] Hoffmann S. Otto C. Kurtz S. Sharma C. M. Khaitovich P. Vogel J. (2009). Fast mapping of short sequences with mismatches, insertions and deletions using index structures. PLoS Comput. Biol. 5:e1000502. 10.1371/journal.pcbi.1000502 19750212 PMC2730575

[B18] Hong J. Qian Z. K. Shen S. Y. Min T. S. Tan C. Xu J. F. (2005). High doses of siRNAs induce eri-1 and adar-1 gene expression and reduce the efficiency of RNA interference in the mouse. Biochem. J. 390, 675–679. 10.1042/BJ20050647 16004606 PMC1199660

[B19] Jia Y. Lisch D. R. Ohtsu K. Scanlon M. J. Nettleton D. Schnable P. S. (2009). Loss of RNA-dependent RNA polymerase 2 (RDR2) function causes widespread and unexpected changes in the expression of transposons, genes, and 24-nt small RNAs. PLoS Genet. 5:e1000737. 10.1371/journal.pgen.1000737 19936292 PMC2774947

[B20] Kennedy S. Wang D. Ruvkun G. (2004). A conserved siRNA-degrading RNase negatively regulates RNA interference in C-*elegans*. Nature 427, 645–649. 10.1038/nature02302 14961122

[B21] Kim J. H. Choi D. Kende H. (2003). The AtGRF family of putative transcription factors is involved in leaf and cotyledon growth in *Arabidopsis*. Plant J. 36, 94–104. 10.1046/j.1365-313X.2003.01862.x 12974814

[B22] Kotakis C. Vrettos N. Kotsis D. Tsagris M. Kotzabasis K. Kalantidis K. (2010). Light intensity affects RNA silencing of a transgene in *Nicotiana benthamiana* plants. BMC Plant Biol. 10:220. 10.1186/1471-2229-10-220 20939918 PMC3017829

[B23] Kozomara A. Griffiths-Jones S. (2014). miRBase: annotating high confidence microRNAs using deep sequencing data. Nucleic Acids Res. 42, D68–D73. 10.1093/nar/gkt1181 24275495 PMC3965103

[B24] Kuhlmann M. Borisova B. E. Kaller M. Larsson P. Stach D. Na J. B. (2005). Silencing of retrotransposons in *Dictyostelium* by DNA methylation and RNAi. Nucleic Acids Res. 33, 6405–6417. 10.1093/nar/gki952 16282589 PMC1283529

[B25] Lamesch P. Berardini T. Z. Li D. Swarbreck D. Wilks C. Sasidharan R. (2012). The *Arabidopsis* Information Resource (TAIR): improved gene annotation and new tools. Nucleic Acids Res. 40, D1202–D1210. 10.1093/nar/gkr1090 22140109 PMC3245047

[B26] Le Trionnaire G. L. Twell D. (2010). Small RNAs in angiosperm gametophytes: from epigenetics to gamete development. Genes Dev. 24, 1081–1085. 10.1101/gad.1936110 20516193 PMC2878646

[B27] Li H. Handsaker B. Wysoker A. Fennell T. Ruan J. Homer N. (2009). The Sequence Alignment/Map format and SAMtools. Bioinformatics 25, 2078–2079. 10.1093/bioinformatics/btp352 19505943 PMC2723002

[B28] Liu D. Song Y. Chen Z. Yu D. (2009a). Ectopic expression of miR396 suppresses GRF target gene expression and alters leaf growth in *Arabidopsis Physiol*. Plant. 136, 223–236. 10.1111/j.1399-3054.2009.01229.x 19453503

[B29] Liu D. Song Y. Chen Z. Yu D. (2009b). Ectopic expression of miR396 suppresses GRF target gene expression and alters leaf growth in *Arabidopsis*. Physiol. Plant. 136, 223–236. 10.1111/j.1399-3054.2009.01229.x 19453503

[B30] Liu X. Yu F. Rodermel S. (2010). An *Arabidopsis* pentatricopeptide repeat protein, SUPPRESSOR OF VARIEGATION7, is required for FtsH-mediated chloroplast biogenesis. Plant Physiol. 154, 1588–1601. 10.1104/pp.110.164111 20935174 PMC2996016

[B31] Mallory A. Vaucheret H. (2010). Form, function, and regulation of ARGONAUTE proteins. Plant Cell 22, 3879–3889. 10.1105/tpc.110.080671 21183704 PMC3027166

[B32] Marker C. Zemann A. Terhorst T. Kiefmann M. Kastenmayer J. P. Green P. (2002). Experimental RNomics: identification of 140 candidates for small non-messenger RNAs in the plant *Arabidopsis thaliana*. Curr. Biol. 12, 2002–2013. 10.1016/S0960-9822(02)01304-0 12477388

[B33] Martinez de Alba A. E. Elvira-Matelot E. Vaucheret H. (2013). Gene silencing in plants: a diversity of pathways. Biochim. Biophys. Acta 1829, 1300–1308. 10.1016/j.bbagrm.2013.10.005 24185199

[B34] Matzke M. Kanno T. Huettel B. Daxinger L. Matzke A. J. M. (2007). Targets of RNA-directed DNA methylation. Curr. Opin. Plant Biol. 10, 512–519. 10.1016/j.pbi.2007.06.007 17702644

[B35] Meyer R. C. Kusterer B. Lisec J. Steinfath M. Becher M. Scharr H. (2010). QTL analysis of early stage heterosis for biomass in *Arabidopsis*. Theor. Appl. Genet. 120, 227–237. 10.1007/s00122-009-1074-6 19504257 PMC2793381

[B36] Meyer R. C. Torjek O. Becher M. Altmann T. (2004). Heterosis of Biomass production in *Arabidopsis.* Establishment during early development. Plant Physiol. 134, 1813–1823. 10.1104/pp.103.033001 15064384 PMC419853

[B37] Meyer R. C. Witucka-Wall H. Becher M. Blacha A. Boudichevskaia A. Dörmann P. (2012). Heterosis manifestation during early *Arabidopsis* seedling development is characterized by intermediate gene expression and enhanced metabolic activity in the hybrids. Plant J. 71, 669–683. 10.1111/j.1365-313X.2012.05021.x 22487254

[B38] Milborrow B. V. (1998). A biochemical mechanism for hybrid vigour. J. Exp. Bot. 49, 1063–1071. 10.1093/jxb/49.324.1063

[B39] Ng D. W. K. Lu J. Chen Z. J. (2012). Big roles for small RNAs in polyploidy, hybrid vigor, and hybrid incompatibility. Curr. Opin. Plant Biol 15, 154–161. 10.1016/j.pbi.2012.01.007 22326630

[B40] Poorter H. Lewis C. (1986). Testing differences in relative growth-rate-a method avoiding curve fitting and pairing. Physiol. Plant. 67, 223–226. 10.1111/j.1399-3054.1986.tb02447.x

[B41] Ramachandran V. Chen X. (2008). Degradation of microRNAs by a family of exoribonucleases in *Arabidopsis*. Science 321, 1490–1492. 10.1126/science.1163728 18787168 PMC2570778

[B42] Ratcliff F. Harrison B. D. Baulcombe D. C. (1997). A similarity between viral defense and gene silencing in plants. Science 276, 1558–1560. 18610513 10.1126/science.276.5318.1558

[B43] Rubio-Somoza I. Weigel D. (2011). MicroRNA networks and developmental plasticity in plants. Trends Plant Sci. 16, 258–264. 10.1016/j.tplants.2011.03.001 21466971

[B44] Ruwe H. Kupsch C. Teubner M. Schmitz-Linneweber C. (2011). The RNA-recognition motif in chloroplasts. J. Plant Physiol. 168, 1361–1371. 10.1016/j.jplph.2011.01.012 21330002

[B45] Ruwe H. Schmitz-Linneweber C. (2012). Short non-coding RNA fragments accumulating in chloroplasts: footprints of RNA binding proteins? Nucleic Acids Res. 40, 3106–3116. 10.1093/nar/gkr1138 22139936 PMC3326302

[B46] Schubert D. Lechtenberg B. Forsbach A. Gils M. Bahadur S. Schmidt R. (2004). Silencing in *Arabidopsis* T-DNA transformants: the predominant role of a gene-specific RNA sensing mechanism versus position effects. Plant Cell 16, 2561–2572. 10.1105/tpc.104.024547 15367719 PMC520955

[B47] Shen H. He H. Li J. Chen W. Wang X. Guo L. (2012). Genome-wide analysis of DNA methylation and gene expression changes in two *Arabidopsis* ecotypes and their reciprocal hybrids. Plant Cell 24, 875–892. 10.1105/tpc.111.094870 22438023 PMC3336129

[B48] Thimm O. Blasing O. Gibon Y. Nagel A. Meyer S. Kruger P. (2004). MAPMAN: a user-driven tool to display genomics data sets onto diagrams of metabolic pathways and other biological processes. Plant J. 37, 914–939. 10.1111/j.1365-313X.2004.02016.x 14996223

[B49] Vaucheret H. Fagard M. (2001). Transcriptional gene silencing in plants: targets, inducers and regulators. Trends Genet. 17, 29–35. 10.1016/S0168-9525(00)02166-1 11163919

[B50] Walter A. Scharr H. Gilmer F. Zierer R. Nagel K. A. Ernst M. (2007). Dynamics of seedling growth acclimation towards altered light conditions can be quantified via GROWSCREEN: a setup and procedure designed for rapid optical phenotyping of different plant species. New Phytol. 174, 447–455. 10.1111/j.1469-8137.2007.02002.x 17388907

[B51] Wang Q. L. Li Z. H. (2007). The functions of microRNAs in plants. Front. Biosci. 12:3975–3982. 10.2741/2364 17485351

[B52] Wilkins C. Dishongh R. Moore S. C. Whitt M. A. Chow M. Machaca K. (2005). RNA interference is an antiviral defence mechanism in *Caenorhabditis elegans*. Nature 436, 1044–1047. 10.1038/nature03957 16107852

[B53] Winter D. Vinegar B. Nahal H. Ammar R. Wilson G. V. Provart N. J. (2007). An “Electronic Fluorescent Pictograph” browser for exploring and analyzing large-scale biological data sets. PLoS ONE 2:e718. 10.1371/journal.pone.0000718 17684564 PMC1934936

[B54] Yang X. C. Purdy M. Marzluff W. F. Dominski Z. (2006). Characterization of 3′ hExo, a 3′ exonuclease specifically interacting with the 3′ end of histone mRNA. J. Biol. Chem. 281, 30447–30454. 10.1074/jbc.M602947200 16912046

[B55] Zimmermann P. Hirsch-Hoffmann M. Hennig L. Gruissem W. (2004). GENEVESTIGATOR. *Arabidopsis* microarray database and analysis toolbox. Plant Physiol. 136, 2621–2632. 10.1104/pp.104.046367 15375207 PMC523327

