# Supplementary figures and images for: Overexpression of Arabidopsis thaliana ERI, the homolog of C. elegans Enhancer of RNAinterference, leads to enhanced growth
Source: Front Plant Sci. 2015 Jul 22;6:531. doi: 10.3389/fpls.2015.00531 (PMC4510415; doi:10.3389/fpls.2015.00531)

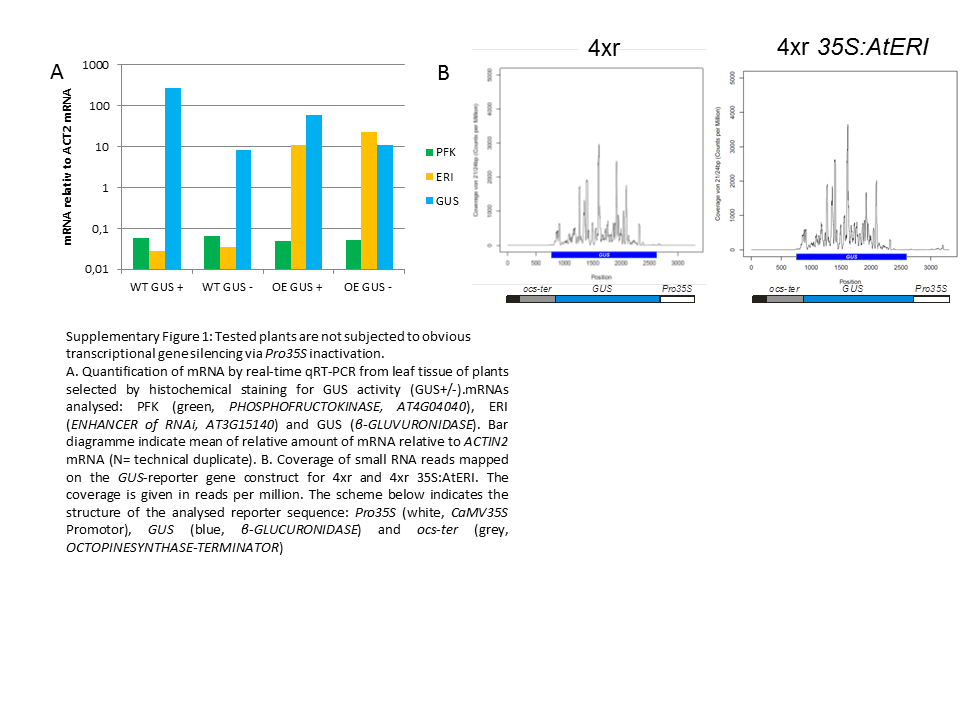

Supplement: Supplementary file 4 [file Image1.TIF]
